# Modification of Cry4Aa toward Improved Toxin Processing in the Gut of the Pea Aphid, *Acyrthosiphon pisum*

**DOI:** 10.1371/journal.pone.0155466

**Published:** 2016-05-12

**Authors:** Michael A. Rausch, Nanasaheb P. Chougule, Benjamin R. Deist, Bryony C. Bonning

**Affiliations:** Department of Entomology, Iowa State University, Ames, Iowa, United States of America; CSIRO, AUSTRALIA

## Abstract

Aphids are sap-sucking insects (order: Hemiptera) that cause extensive damage to a wide range of agricultural crops. Our goal was to optimize a naturally occurring insecticidal crystalline (Cry) toxins produced by the soil-dwelling bacterium *Bacillus thuringiensis* for use against the pea aphid, *Acyrthosiphon pisum*. On the basis that activation of the Cry4Aa toxin is a rate-limiting factor contributing to the relatively low aphicidal activity of this toxin, we introduced cathepsin L and cathepsin B cleavage sites into Cry4Aa for rapid activation in the aphid gut environment. Incubation of modified Cry4Aa and aphid proteases *in vitro* demonstrated enhanced processing of the toxin into the active form for some of the modified constructs relative to non-modified Cry4Aa. Aphids fed artificial diet with toxin at a final concentration of 125 μg/ml showed enhanced mortality after two days for one of the four modified constructs. Although only modest toxin improvement was achieved by use of this strategy, such specific toxin modifications designed to overcome factors that limit aphid toxicity could be applied toward managing aphid populations via transgenic plant resistance.

## Introduction

Aphids can cause extensive economic losses to agricultural crops, with some U.S. $1.6 billion in costs attributed to the soybean aphid, *Aphis glycines* in the United States alone [1]. Yield losses occur through direct feeding, transmission of numerous plant viruses [2] and from aphid honeydew which provides a medium for fungal growth [3]. Current aphid management relies primarily on the application of chemical insecticides that may have negative environmental consequences and to which aphids can rapidly develop resistance [4, 5].

Transgenic crops incorporating insecticidal crystal toxins (Cry) isolated from the bacterium *Bacillus thuringiensis* have been applied for management of other insect pests [6–11], resulting in increased yields, and decreased use of chemical insecticides [9–11]. However, exposure to Bt toxins results in little or no mortality in aphids [12–14]. Aphids (Hemiptera) use piercing-sucking mouthparts to feed on plant phloem resulting in minimal natural exposure to Bt toxins which are present in the soil and on leaf surfaces. Hence, there has likely been little natural selection for toxicity against Hemiptera [15].

Following ingestion, Cry toxins are solubilized and become activated by insect gut proteases [16]. The activated toxin binds to receptors on the insect gut epithelium. Conformational changes in the toxin result in insertion into the gut epithelial membrane, pore formation, and epithelial cell lysis through osmotic disruption [17–19]. In susceptible species, toxicity results in gut paralysis, reduced feeding, and extensive damage to epithelial cells, ultimately resulting in death of the insect [20–22].

Similar to some coleopteran species that are susceptible to Cry toxins, the aphid gut is mildly acidic in the stomach and neutral in the midgut and hindgut, with the major gut proteases being cysteine proteases of the cathepsin L and cathepsin B type [23, 24]. In contrast, in susceptible lepidopteran and dipteran insects, the primary gut proteases are serine proteases which are active at alkaline pH [16, 25]. Cry toxins with activity against these groups are efficiently processed in this alkaline gut environment [26, 27].

Activation of Cry4Aa prior to insect feeding results in increased activity against the pea aphid, *Acyrthosiphon pisum* [28], indicating that toxin activation is a limiting step in Cry toxicity against aphids [28]. In addition it has been suggested that Cry toxins can be modified to achieve toxin activation in the gut of less susceptible insects: Insertion of a chymotrypsin G site between α-helices 3 and 4 of domain I of Cry3A, resulted in cleavage at this site by gut proteases, enhanced toxin activation and increased toxicity in the western corn rootworm, *Diabrotica virgifera virgifera* [29].

Cry4Aa derived from *Bacillus thuringiensis* subsp *israelensis* is a member of the three-domain Cry toxin family. Cry4Aa is toxic to multiple mosquito species [16, 30], and the crystal structure has been resolved. For the three-domain Cry toxins, domain I is involved in pore formation in the insect gut [30–34]. Domain II contains residues involved in receptor binding of target insects [30, 35–37]. Domain III is also implicated in receptor binding as well as in maintenance of toxin stability [30, 37–39]. Cry4Aa is produced as a 130-kDa protoxin that is converted into protease-resistant 45 and 20 -kDa fragments through a 60–65 -kDa intermediate. The 45 and 20-kDa fragments are generated through intramolecular cleavage and re-associate by electrostatic interactions to form an active toxin monomer [30], hence both fragments are required for toxicity [40]. An *in silico* study of the active toxin monomers indicates that three monomers associate via domain I to form a trimer, with several helices in domain I forming a pore [41].

In this study, we inserted cathepsin L and B cleavage sites into Cry4Aa to test the hypothesis that these sites will facilitate activation of Cry4Aa in the aphid gut resulting in improved toxicity against the pea aphid. Activation of native and modified Cry4Aa was visualized after exposure to pea aphid proteases both *in vitro* and *in vivo*. In addition, feeding assays of native and modified toxins were conducted with pea aphids to test for improvement in toxicity. The targeted modification approach adopted for this study to overcome specific factors limiting aphid toxicity may be useful for the production of aphid resistant transgenic plants, providing an additional management tool for damaging aphid populations.

## Materials and Methods

### Construction of Modified Cry4Aa-S1

**Fig 1:**
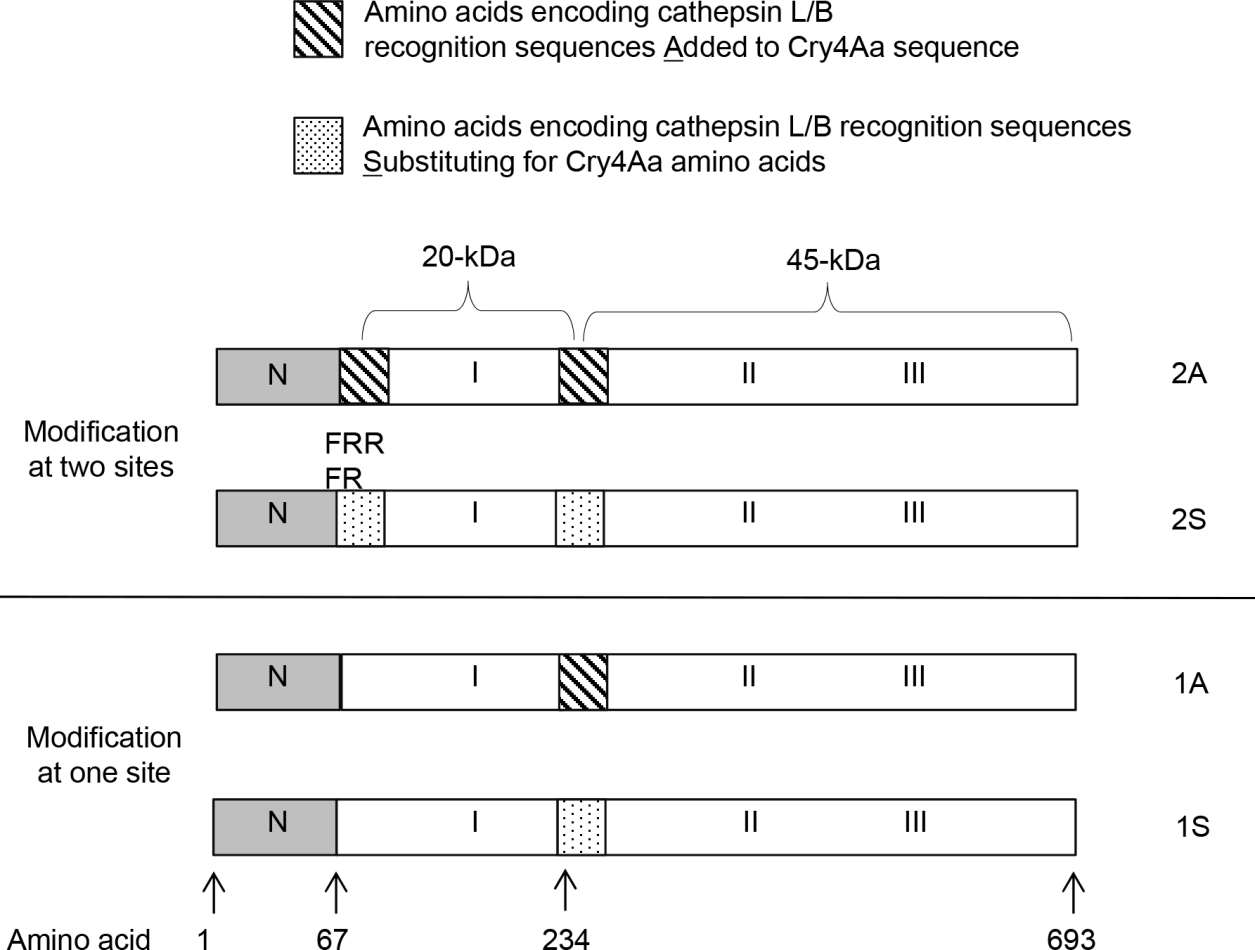
Engineering of Cry4Aa with cathepsin L and B cleavage sites. Amino acid sequences that are recognized by cathepsin L and B proteases (FR and RR respectively) were added to Cry4Aa or replaced existing amino acids, at two locations. Two additional Cry4Aa constructs were modified at the second region only. See Table 1 for details of the modified toxins produced.

The toxin gene *cry4Aa-S1* [42], was used for modification for enhanced cathepsin-mediated activation of Cry4Aa. The codon sequence and G+C content of *Cry4Aa-S1* was modified for *E*. *coli* expression, without alteration of the amino acid sequence relative to wild type Cry4Aa [42]. CryAa-S1 produces the intermediate 65 kDa protein rather than the full length 130 kDa protoxin [42]. To introduce the cathepsin L and cathepsin B cleavage sites (FRR and FR, respectively) into the *cry4Aa-S1* gene, we used PCR to introduce the modified sequences, and overlap extension polymerase chain reaction (OE-PCR) to splice DNA fragments together. For the construct Cry4Aa 2A, nucleotide sequences encoding the three amino acids ‘FRR’ and two amino acids ‘FR’ were added at distinct sites in domain I of Cry4Aa-S1, after amino acid 67 and 234 respectively: the sequence introduced after amino acid 234 was immediately upstream of an arginine codon, such that FRR was also encoded at this site (Fig 1). A second construct, Cry4Aa 2S with FRR and FR replacing (rather than adding to) amino acids in Cry4Aa (I65F, D66R, S67R, and N233F, N234R) was also made. The second modification site (location 234) is the region where the 45 and 20 -kDa fragments are separated in Cry4Aa. Two additional constructs (Cry4Aa 1A and Cry4Aa 1S) with added or substituted sequences encoding FR at this second site only were made, for a total of four modified constructs of Cry4Aa (Table 1).

**Table 1:** Modification of Cry4Aa with cathepsin L/B-specific cleavage sites. For construct names, A is used when amino acids were added, and S when amino acids were substituted at one or two sites as indicated. Sites modified are numbered according to amino acid numbers in Cry4Aa.

Construct	Type	Modification	Amino Acid Sequence
Cry4Aa	Wild type	-	63:TFIDSGEL
			231:LKNNRQ
Cry4Aa 2A	Addition FRR and FR	Between S67 and G68;	TFIDS**FRR**GEL
		Between N234 and R235	LKNN**FR**RQF
Cry4Aa 2S	Substitution FRR and FR	Replaced I65F, D66R, S67R	TF**FRR**GEL
		Replaced N233F, N234R	LK**FR**RQF
Cry4Aa 1A	Addition FR	Between N234 and R235	LKNN**FR**RQF
Cry4Aa 1S	Substitution FR	Replaced N233F, N234R	LK**FR**RQF

Primers designed to add or substitute cathepsin L/B sequences (Table 2) were incubated with the *cry4Aa-S1* gene in separate PCR reactions using Phusion Hot Start II DNA polymerase (Fermentas UAB, subsidiary of Thermo Fisher Scientific Inc.). Amplified products were visualized by agarose gel and ethidium bromide staining using standard protocols, and bands of expected product size were excised and purified using a QIAquick gel extraction kit (Qiagen, Venlo, Limburg, Netherlands) according to the manufacturer’s protocol. Purified DNA fragments were quantified with a spectrophometer (Nanodrop 2000c spectrophotometer, Thermo Scientific, Waltham, MA). Appropriate fragments to produce the addition and substitution *cry4Aa-S1* modified genes were incubated together with end primers (Tables 2 and 3) encoding restriction sites for *Eco*R1 and *Bam*H1, and Phusion Hot Start II DNA polymerase. Amplified products were excised and purified as described above, cloned into pGEX-2T using the restriction sites *Eco*R1 and *Bam*H1, and transformed into BL21 Z competent *E*. *coli* cells. Native (non-modified) Cry4Aa-S1 was also cloned and transformed as described for the modified constructs. Positive clones for each modified construct were identified by colony PCR. The inserts in selected clones were sequenced by the Iowa State University DNA Facility to confirm the sequence and frame of the modified *cry4Aa-S1* genes.

**Table 2:** Construction of modified Cry4Aa constructs. Primers used in OE-PCR reactions. Primers 0F and 5R contain *Bam H1* and *Eco R1* restriction sites respectively (shown in bold) for use in cloning.

Primer Number	Primer Sequence
0F	5’ TA**GGATCC**ATGAACCCGTACCAAAAC 3’
1F	5’ATGGATCCATGAACCCGTATCAAAATAAAAACGA 3’
2F	5’ATTCGAAACGTTCATCGATTCATTCCGTCGTGGTGAACTGTCGGCATACACCATC 3’
3F	5’TCGAAGCGTATCTGAAAAACAATTTCCGTCGTCAGTTCGACTATCTGGAAC 3’
4F	5’ACGGTGGTGACTTCGAAACGTTCTTCCGTCGTGGTGAACTGTCGGCATACACCATC 3’
5F	5’ TGAAATTCGAAGCGTATCTGAAATTCCGTCGTCAGTTCGACTATCTGGAAC 3’
1R	5’ TGAATCGATGAACGTTTCGAAGT 3’
2R	5’ ATTGTTTTTCAGATACGCTTCGA 3’
3R	5’ GAACGTTTCGAAGTCACCACCGT 3’
4R	5’ TTTCAGATACGCTTCGAATTTCA 3’
5R	5’ TAG**AATT**CTCACACGGTTTCCAGTTTTTG 3’

**Table 3:** Construction of modified Cry4Aa constructs. OE-PCR reactions with separate reactions indicated by square brackets.

Cry4Aa Construct	OE-PCR Reactions
2A	[0F + [1F+1R] [2F+2R] [3F+5R] + 5R]
2S	[0F + [1F+3R] [4F+4R] [5F+5R] + 5R]
1A	[0F + [1F+2R] [3F+5R] + 5R]
1S	[0F + [1F+4R] [5F+5R] + 5R]

### Expression and purification of modified Cry4Aa

Modified and wild type Cry4Aa-S1 toxins were expressed using pGEX-2T and purified as glutathione S-transferase (GST)–toxin fusion proteins using standard procedures. Expression of the GST-Cry4Aa-S1 fusions was induced with 0.06 mM isopropyl β-D-1-thiogalactopyranoside (IPTG) and incubated at 20°C with shaking (220 rpm) for 5 hours. Cry4Aa was liberated from the Glutathione Sepharose ® (GSH) 4B beads (GE Healthcare Bio-Sciences AB, Uppsala, Sweden), with 50 units of thrombin and incubation at 4°C overnight. Five 500-μl fractions of purified Cry4Aa-S1 toxin were collected and proteins visualized following separation on 12% sodium dodecyl sulfate polyacrylamide gels (SDS-PAGE), alongside samples of the cell lysate, flow through, and a wash of the column following fraction collection. Fractions with bands of the correct size were pooled and concentrated by using Amicon Ultra-0.5 Centrifugal Filter Devices with a 3-kDa cut-off (Merck Milipore Ltd., Co Cork, IRL). For mosquito and aphid bioassays, purified toxin buffer was exchanged with 10 mM Tris pH 7.5 using Amicon Ultra-0.5 Centrifugal Filter Devices (3-kDa). Protein concentration was determined using a Bradford Assay with BSA as a standard.

### Impact of modifications on Cry4Aa toxicity

Larvae of *Culex pipiens* (3 day old) were used in bioassays to address whether modifications made impacted Cry4Aa toxicity. Cry4Aa and modified toxins (5μg/ml) were tested in 24-well culture plates, with 4 technical replicates of six larvae per well in a volume of 2 ml. To control for the potential impact of Tris buffer on larval survival, a volume of Tris buffer (10 mM, pH 7.5) equivalent to the volume of toxin added was added to control wells. Plates were incubated at 28°C with 75% humidity and an 18:6 light:dark photoperiod. Mortality of larvae was recorded every 24 h, and the assay was run for 2 days. The bioassay was replicated twice.

### Trypsin digestion of Cry4Aa and modified toxins

Native Cry4Aa (Cry4Aa-S1) was digested with trypsin (Sigma-Aldrich Co. LLC., St. Louis, MO) at different concentrations (up to 20% w/w) and for different periods of time (up to 24 hours) at 37°C to examine cleavage of the 60 kDa intermediate protein [40]. Digestion products were examined on Coomassie blue-stained 12% SDS-PAGE gels. To confirm the identity of the 60 kDa intermediate, proteins were transferred to PVDF membrane and submitted to the Iowa State University Protein Facility for N-terminal sequencing of the 60 kDa protein band. The structural stability of modified Cry4Aa-S1 was examined by trypsin digestion (5%) of 200 ng toxin for 3 hours at 37°C. Digestion products were examined by SDS-PAGE.

### Processing of modified toxins by aphid gut cathepsins under *in vitro* conditions

Pea aphids were maintained in an environmental chamber (L:D 24:0 h, 22°C, 70% RH) on fava bean (*Vicia faba*; Peaceful Valley Farm and Garden Supply, Grass Valley, CA). Aphids were placed in a dissecting well containing 50 μl of 30 mM sodium acetate pH 6.0. Ten aphid guts were dissected in a 30-minute time period, pooled and snap frozen in liquid nitrogen. A total of 250 aphid guts were prepared in this way. Samples were thawed on ice and pooled. Gut tissue was homogenized in an Eppendorf tube using a pestle and centrifuged at 16,000 *g* for 25 minutes at 4°C. The supernatant was drawn off and concentrated using Amicon Ultra-0.5 Centrifugal Filter Devices (3-kDa) and labeled as the lumen fraction. The gut pellet was resuspended in 200 μl of 30 mM sodium acetate pH 6.0 and labeled as the membrane fraction. The protein concentration of each fraction was determined by Bradford Assay using BSA as a standard. Both fractions were snap frozen in liquid nitrogen and stored at -80°C.

To examine the processing of the native and modified Cry4Aa-S1 toxins under *in vitro* conditions, a 5:1 ratio (gut sample protein: toxin, w/w) for the lumen and membrane gut fractions in a volume of 20 μl was used. A total of 1 μg of gut lumen or membrane sample was used for each reaction. Membrane and lumen samples were incubated for 1 hour at room temperature in the presence or absence of cysteine protease activators (ethylenediaminetetraacetic acid, EDTA and cysteine added to final concentrations of 3 mM). A total of 200 ng of native or modified Cry4Aa-S1 toxin was then added to each sample and incubated at room temperature while shaking at 250 rpm for 3 hours. Negative controls included modified toxins that were incubated in the absence of gut proteins or activators. Reactions were stopped with 5X Laemmli buffer and heated to 100°C for 5 min. Western blot visualization of processed toxin was conducted using polyclonal Cry4Aa antiserum (1:5000 dilution). This antiserum was generated in rabbits (New Zealand White) by injecting bacterially expressed Cry4Aa (Iowa State University Hybridoma Facility). The secondary antibody was HRP-conjugated anti-rabbit IgG (1:5000). Immunoreactive bands were detected by incubating the nitrocellulose membrane in Hyglo Chemiluminescent HRP detection reagent for 1 minute and exposure to X-ray film using standard procedures. Solubilized native and modified toxins (200 ng) were used as negative control samples for protease digestion. Trypsin-activated native Cry4Aa-S1 (200 ng) activated with 5% trypsin (Sigma-Aldrich Co. LLC., St. Louis, MO) for 3 hours at 37°C was used as a positive control.

### Aphid toxicity assays

For membrane feeding assays, filter-sterilized 2X complete aphid artificial diet [43] diluted with 10 mM Tris pH 7.5 was placed on Parafilm® stretched thinly across a 3-cm cell culture plate with a 1-cm hole and covered with a second layer of Parafilm®. Native and modified Cry4Aa-S1 in 10 mM Tris pH 7.5 were mixed separately with complete aphid diet to a final concentration of 125 μg/ml. A volume of 100 μl of toxin/diet mixture was added to each feeding dish. A total of 15 second-instar pea aphids were transferred to each plate and incubated at 22°C. Aphid mortality was scored every 24 hours for 4 days. Complete aphid diet diluted 10 mM Tris pH 7.5 was included as a negative control and complete diet with trypsin-activated Cry4Aa-S1 toxin (prepared as described above) was included as a positive control. Trypsin was removed following toxin activation using benzamidine sepharose beads (GE Healthcare Bio Sciences, AB, Sweden) prior to use in feeding assays. Three replicate feeding assays were conducted for each control and treatment group, except for the trypsin control (two replicates). A binomial comparison was used for analysis of feeding assay data. Calculated z-scores, representing the number of standard deviations above or below the mean were used to generate p-values for each comparison. Generated p-values from the binomial comparison analysis were used in a multiple comparison analysis. For multiple comparisons a Bonferroni adjustment was used to calculate the revised threshold (0.05 threshold divided by 21 comparisons) of p<0.002 to indicate significant differences.

### Impact of modified toxins on pea aphid gut

Second instar pea aphids were fed on a single concentration (125μg/ml) of native Cry4Aa, modified Cry4Aa that showed increased toxicity, or 10 mM Tris pH 7.5 in complete artificial diet. Aphid feeding dishes and diet were set up as described for aphid toxicity assays. A total of 12 aphids per treatment were allowed to feed for 24 and 48 hours at 22°C. Following feeding the head, legs, and cornicles were removed and were immediately fixed in 2% v/v paraformaldehyde, 2.5% v/v glutaraldehyde, and 0.05 M cacoldylate. Fixed aphids were submitted to the ISU Microscopy and NanoImaging Facility for examination of midguts by light microscopy to detect disruption of aphid gut tissue following feeding on Cry4Aa toxins.

### Processing of modified toxins by aphid gut cathepsins under *in vivo* conditions

Protocols for these experiments were based on those of Li et al. [27]. Native Cry4Aa-S1 and modified Cry4Aa toxins with increased aphicidal activity were fed to aphids at a concentration of 300 ng/μl in complete artificial diet, with 2 μl blue food coloring per 100 μl of diet. Nine feeding dishes were set up for each treatment as described above with 20 third-instar larvae transferred to each plate and incubated at 22°C overnight. A total of 60 aphid guts showing blue coloration were excised in 20 μl 10 mM Tris pH 7.5 for the native- and modified- toxin-fed groups, and snap frozen in liquid nitrogen. Aphid guts were thawed on ice, homogenized with a pestle and centrifuged at 16,000 *g* for 25 minutes at 4°C. Lumen and membrane fractions were isolated and western blot visualization of 60 aphid guts were conducted as described above.

## Results

### Synthesis, expression and toxicity of modified Cry4Aa toxins

**Fig 2:**
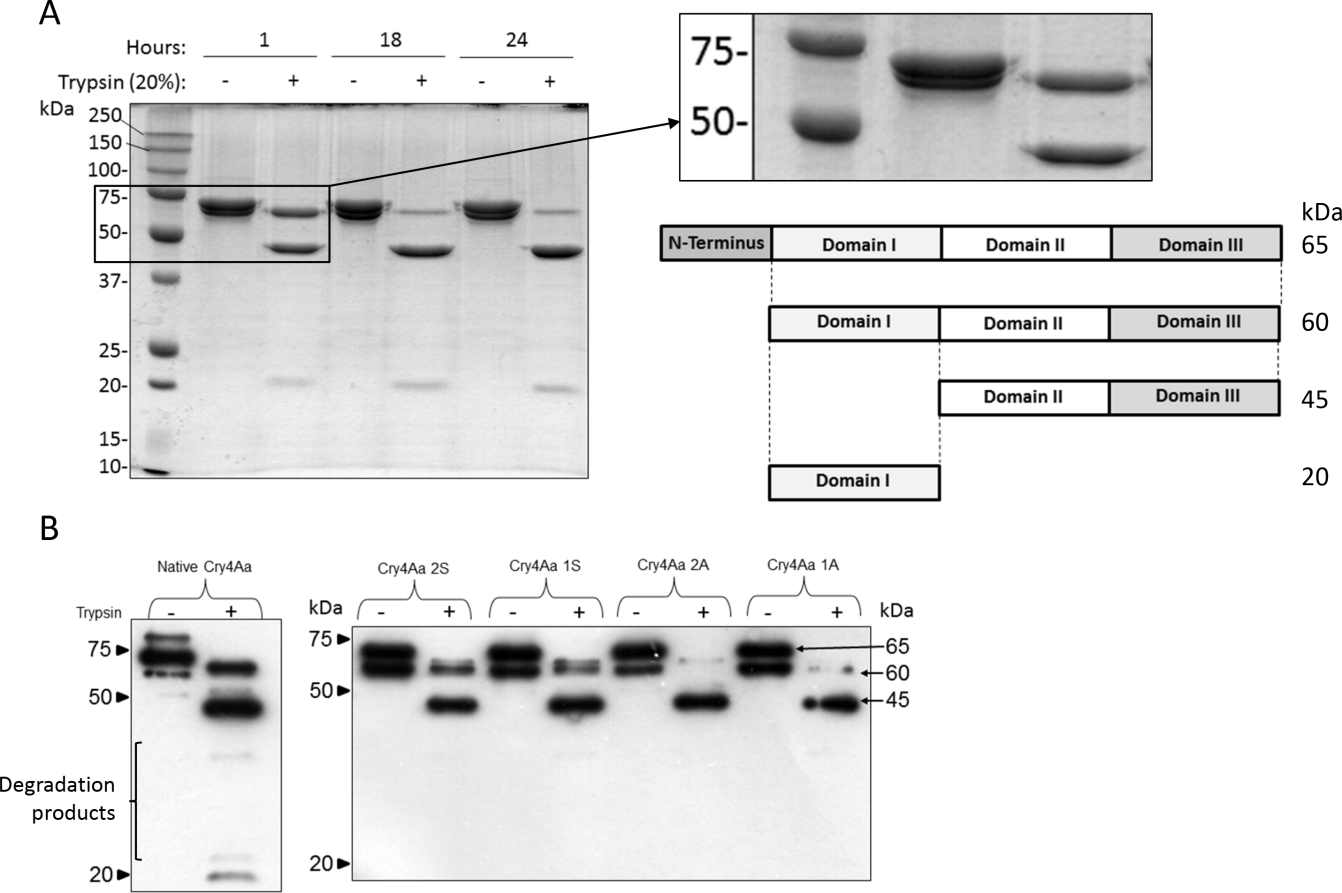
Trypsin activation of native and modified Cry4A toxins. A. Digestion of native Cry4Aa S1 with 20% trypsin for the specified periods of time resulted in bands of 60, 45 and 20 kDa. The composition of these bands is shown in the schematic diagram at right. Only on prolonged digestion with 20% w/w trypsin (18 and 24 hours) did the 60 kDa intermediate band diminish in intensity. The inset shows the presence of two protein bands in the undigested, purified Cry4Aa-S1, which are assumed to be the 65 and 60 kDa proteins resulting from loss of the N-terminal 36 aa. B. Comparison of the trypsin digestion of the native and modified toxins showed variation in the relative band intensities, with significantly less 60 kDa intermediate in the Cry4Aa 1A and 2A digests. Digestion products separated by SDS-PAGE were transferred to PVDF membrane for western blot detection with anti-Cry4Aa IgG.

Sequencing verified that positive clones contained the correct sequences in the correct reading frame for all constructs. Native Cry4Aa toxins were induced and purified as described above, resulting in purified protein of the expected size that reacts positively with Cry4Aa antiserum in western blot. All modified toxins retained toxicity relative to Cry4Aa in mosquito bioassays with *C*. *pipiens* larvae. Mortality of 87 to 100% was seen for all toxins by day 2 of the bioassay, with 12% mortality in the buffer control treatment (data not shown). On exhaustive trypsin digestion (20% trypsin with an 18 hour incubation period) of native Cry4Aa, depletion of the 60 kDa intermediate band was observed (Fig 2A). N-terminal sequencing confirmed that the 60 kDa protein was the Cry4Aa intermediate protein. A 36 amino acid N-terminal sequence was cleaved from the 65 kDa toxin leaving an N-terminal amino acid sequence of the 60 kDa protein of QLLQSTNYKD. Purified modified Cry4Aa constructs appeared as two prominent bands at 60 and 65 -kDa in polyacrylamide gels, both of which were detected by western blot (Fig 2B). Examination of undigested native Cry4Aa in Fig 2A indicates that two bands are present in the undigested toxin sample. Trypsin activation of the modified Cry4Aa toxins resulted in processing with the expected 45 band (but not the 20 kDa band) visible on the western blot (Fig 2B). The native Cry4Aa trypsin digestion blot in Fig 2B was over-exposed to show the 20 kDa protein. Additional degradation products between the 20 and 45 kDa bands were also evident on overexposure of this film, as noted previously [44].

### *In vitro* impacts of pea aphid gut proteases on modified and native Cry4Aa toxins

**Fig 3:**
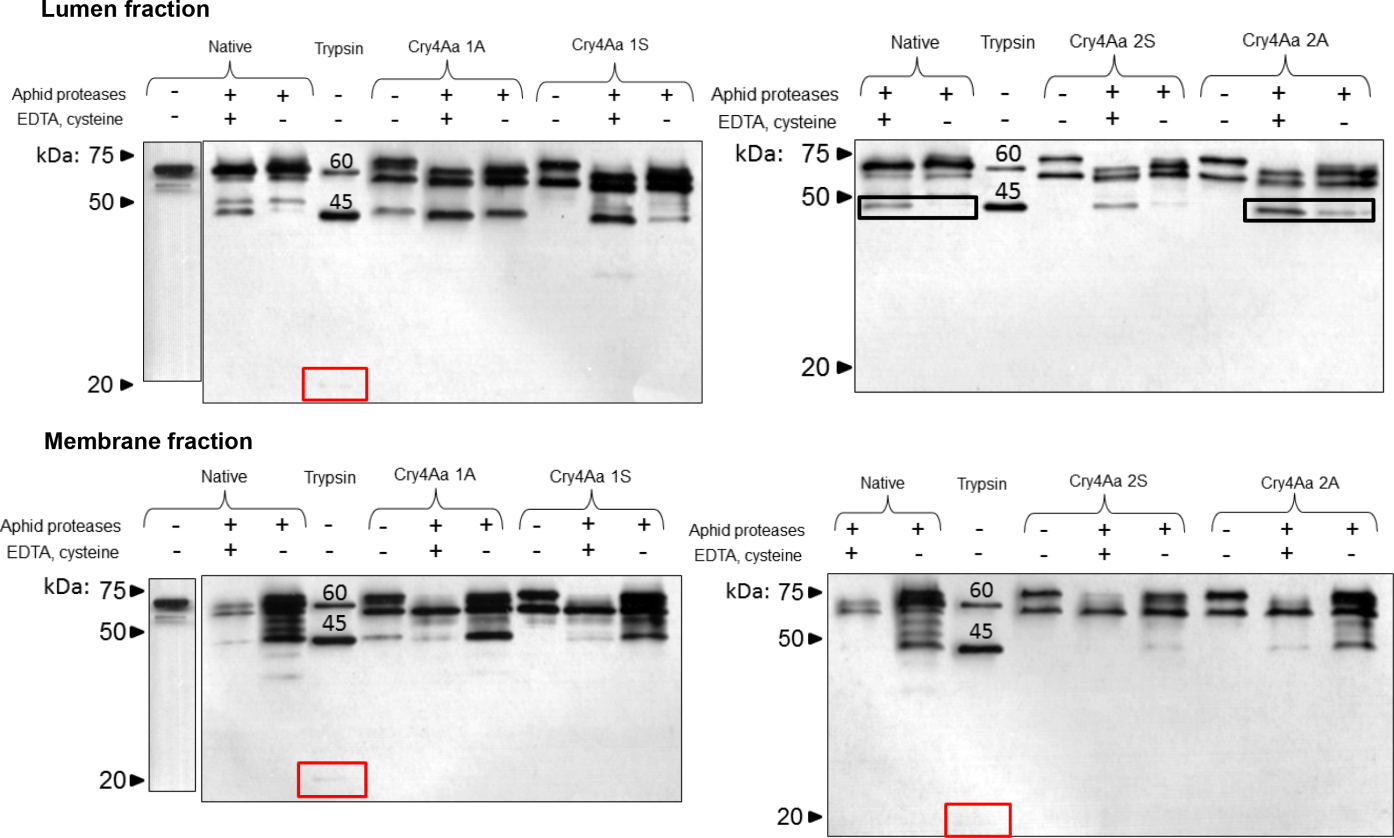
*In vitro* activation of modified Cry4Aa by aphid gut proteases. Toxins were incubated with the lumen or membrane extracts from the pea aphid gut and hydrolyzed products detected by western blot using Cry4Aa antibodies. Toxin activation profiles were generated in the presence or absence of cathepsin activators (EDTA and cysteine, 3 mM) as indicated. Native Cry4Aa with or without digestion with 5% (w/w) trypsin, and modified Cry4Aa not exposed to proteases or activators were included as controls. Native; native Cry4Aa. Trypsin; native Cry4Aa exposed to 5% w/w trypsin, positive control. Boxes show the faint 20 kDa bands seen on trypsin digestion of native Cry4Aa, and comparison of 45 kDa band lumen fraction degradation products of native Cry4Aa and Cry4Aa 2A toxins.

Incubation of negative controls (modified toxins incubated without aphid gut proteases or EDTA and cysteine) resulted in the 60- and 65-kDa bands with no activation to the 45 and 20 kDa proteins observed (Fig 3), except for Cry4Aa 1A which showed a faint 45-kDa band. Exposure of native Cry4Aa toxins to pea aphid lumen gut proteases in the presence of protease activators resulted in partial activation as indicated by the presence of the 45-kDa band. The absence of activators resulted in decreased activation as only a faint 45-kDa band was observed. In contrast the processing of all four modified Cry4Aa constructs by pea aphid lumen gut proteases in the presence of protease activators was enhanced as indicated by a prominent 45-kDa band of similar intensity to the positive control (Cry4Aa digested with 5% trypsin). Cry4Aa 2S showed a similar level of activation to that of the native Cry4Aa. Toxin processing was decreased in the absence of protease activators for all modified toxins except for Cry4Aa 1A which showed a prominent 45-kDa band. The 45-kDa toxin band was barely detectable on exposure of native or modified Cry4Aa toxins to pea aphid membrane gut proteases in the presence of protease activators, indicating over-activation and degradation of the 45-kDa band. Exposure to membrane proteases in the absence of activators revealed several protein bands in addition to the 45-kDa band.

### Aphid toxicity assays

**Fig 4:**
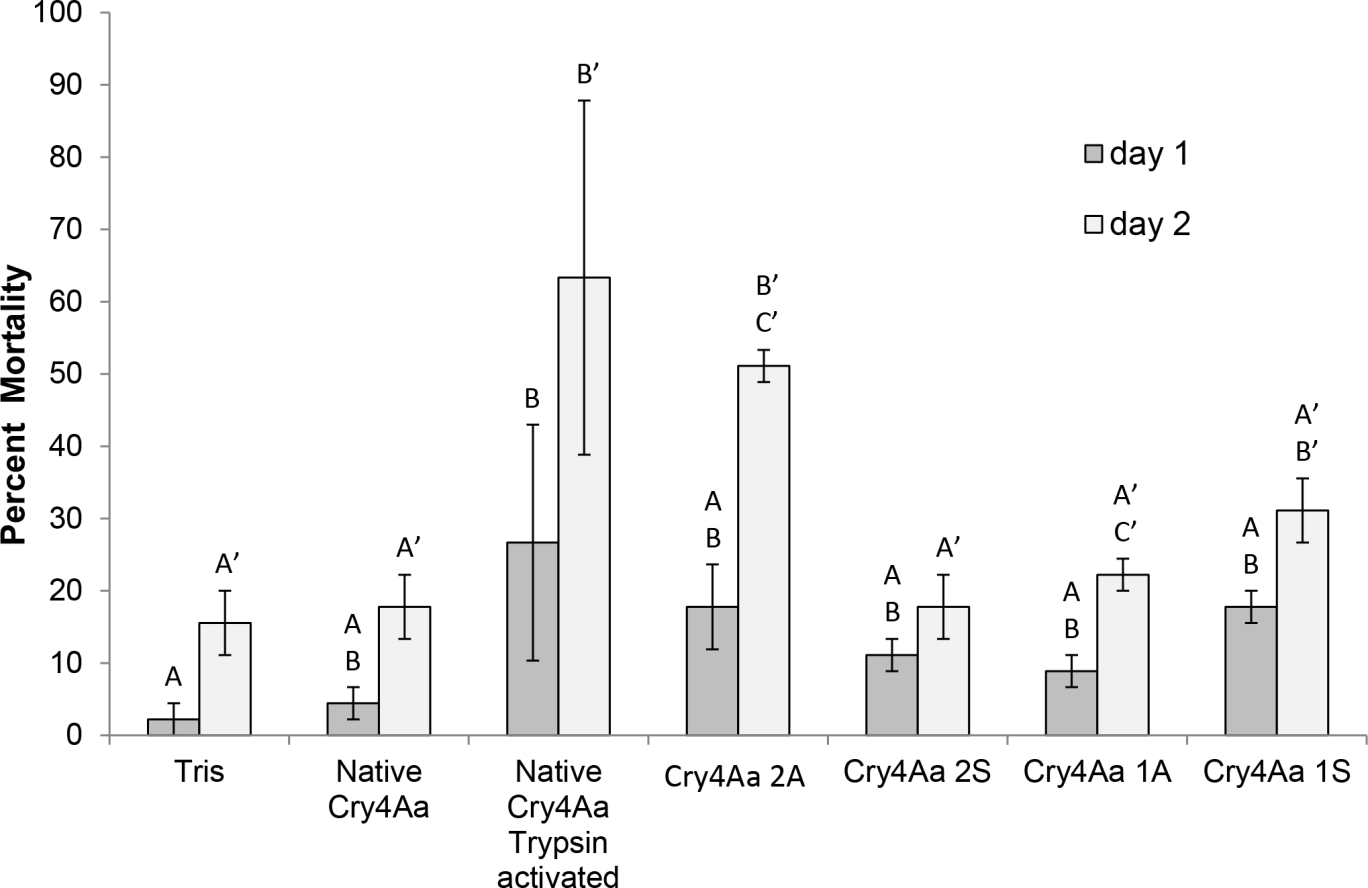
Impact of modified Cry4Aa on aphid survival. Pea aphid mortality (%) after one and two days of feeding on Tris buffer, pH 7.5, native Cry4Aa, native trypsin-activated Cry4Aa, and modified Cry4Aa toxins is shown (mean ± SE). Mortality from treatments with different letters on the same day are significantly different (Bonferroni adjustment, p <0.002 is significantly different) by binomial comparisons, with letters N for day 1 and N’ for day 2.

When making multiple comparisons, a Bonferroni adjustment was used to determine a revised threshold: In the following analyses p<0.002 indicate significant differences between treatments. Low mortality was observed in the Tris (15.6%) and native Cry4Aa (17.8%) controls after two days of feeding (Table 4), in line with previous research that showed that Cry4Aa exhibits low toxicity against pea aphids (Fig 4)[28]. Pea aphids fed on trypsin-activated Cry4Aa showed significantly increased mortality after one day of feeding (21%) relative to Tris (z-score = -3.19, p = 0.0014) but was not greater than that for aphids fed native Cry4Aa (z-score = -2.77, p = 0.006). In contrast trypsin-activated native Cry4Aa showed significantly increased toxicity against the pea aphid after two days of feeding (63%) relative to the Tris (z-score = -4.26, p = 0.00002) and native Cry4Aa treatments (z-score = -4.03, p = 0.0001). Among the modified Cry4Aa toxins, aphids fed Cry4Aa 2A showed similar mortality when compared to Tris (z-score = -2.46, p = 0.014) and native Cry4Aa (z-score = -2.01, p = 0.044) after one day of feeding. However, after two days of feeding, aphids in the Cry4Aa 2A treatment showed significantly increased toxicity (51%) relative to Tris (z-score = -3.58, p = 0.0003), and to native Cry4Aa (z-score = -3.33, p = 0.0009) and was similar to aphid mortality in the trypsin-activated Cry4Aa treatment (z-score = 1.04, p = 0.3) (Fig 4). Mortality in the Cry4Aa 1S treatment was not significantly different from mortality in the native Cry4Aa after one or two days of feeding (day one: z-score = -2.0, p = 0.004; day two: z-score = -1.47, p = 0.14) or trypsin- activated Cry4Aa treatments (day one: z-score = 0.92, p = 0.35; day 2: z-score = 2.74, p = 0.006). Mortality in the Cry4Aa 2S treatment was not significantly different from the Tris (day 1: z-score = -1.7, p = 0.09; day two: z-score = -0.28, p = 0.78) and native Cry4Aa treatments (day 1: z-score = -1.2, p = 0.24); day two: z-score = 0, p = 1). Mortality in the Cry4Aa 1A treatment also was not significantly different from the Tris (day one: z-score = -1.4, p = 0.17; day two: z-score = -0.81, p = 0.42) and native Cry4Aa treatments (day one: z-score = -0.85, p = 0.4); day two: z-score = -0.53, p = 0.60).

**Table 4:** Aphid mortality at 24 and 48 hours after exposure to modified Cry4Aa. Average % daily mortality during 48 hour feeding assay (% average mortality, SE: standard error).

	Day 1		Day 2	
Treatment	% Mortality	SE	% Mortality	SE
Tris	2.2	2.2	15.6	4.4
Native	4.4	2.2	17.8	4.4
Trypsin-activated native	26.7	16.3	63.3	24.5
Cry4Aa 2A	17.8	5.9	51.1	2.2
Cry4Aa 2S	11.1	2.2	17.8	4.4
Cry4Aa 1A	8.9	2.2	22.2	2.2
Cry4 Aa 1S	17.8	2.2	31.1	4.4

**Fig 5:**
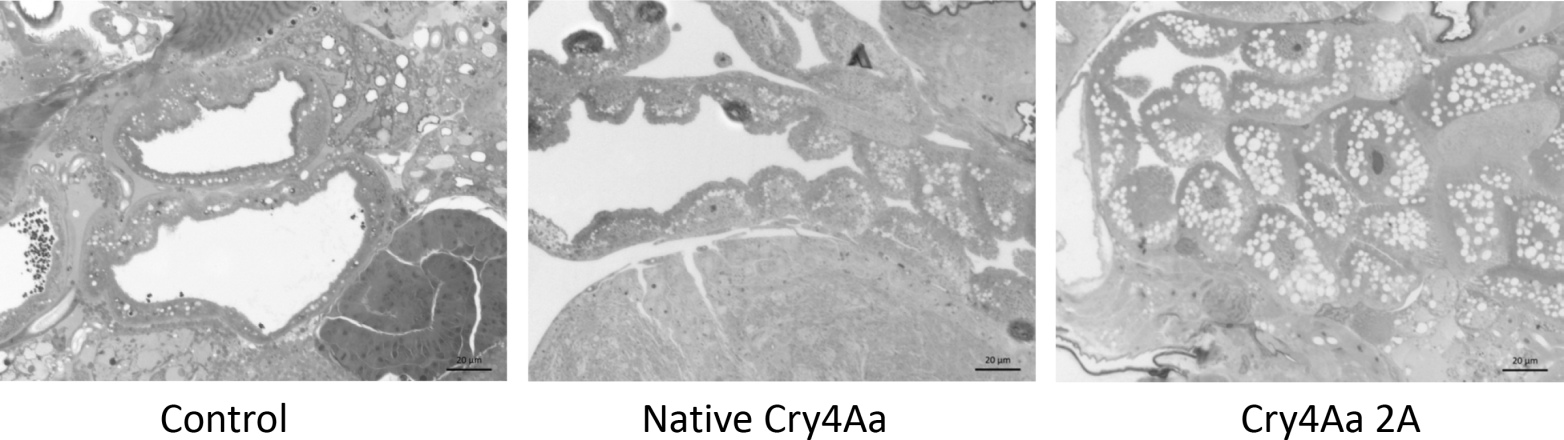
Impact of Cry4Aa 2A on the aphid midgut epithelium. Light microscope images of guts from aphids fed for 24 h on 10 mM Tris pH 7.5 (control), native Cry4Aa or Cry4Aa 2A in the same buffer. The control image shows a healthy gut epithelium, while epithelia from the toxin treatments show mild (native Cry4Aa) and severe (Cry4a 2A) loss of integrity. Epithelial cells swell, become highly vacuolated, and lyse following toxin binding.

Consistent with the bioassay results, light microscopy revealed moderate and severe damage to the pea aphid gut epithelium following 24 h exposure to native Cry4Aa and to Cry4Aa 2A respectively (Fig 5). Epithelial cells were swollen and highly vacuolated in the two toxin treatments, with the gut lumen significantly reduced in the Cry4Aa 2A treatment.

### Modified toxin processing in the pea aphid gut

**Fig 6:**
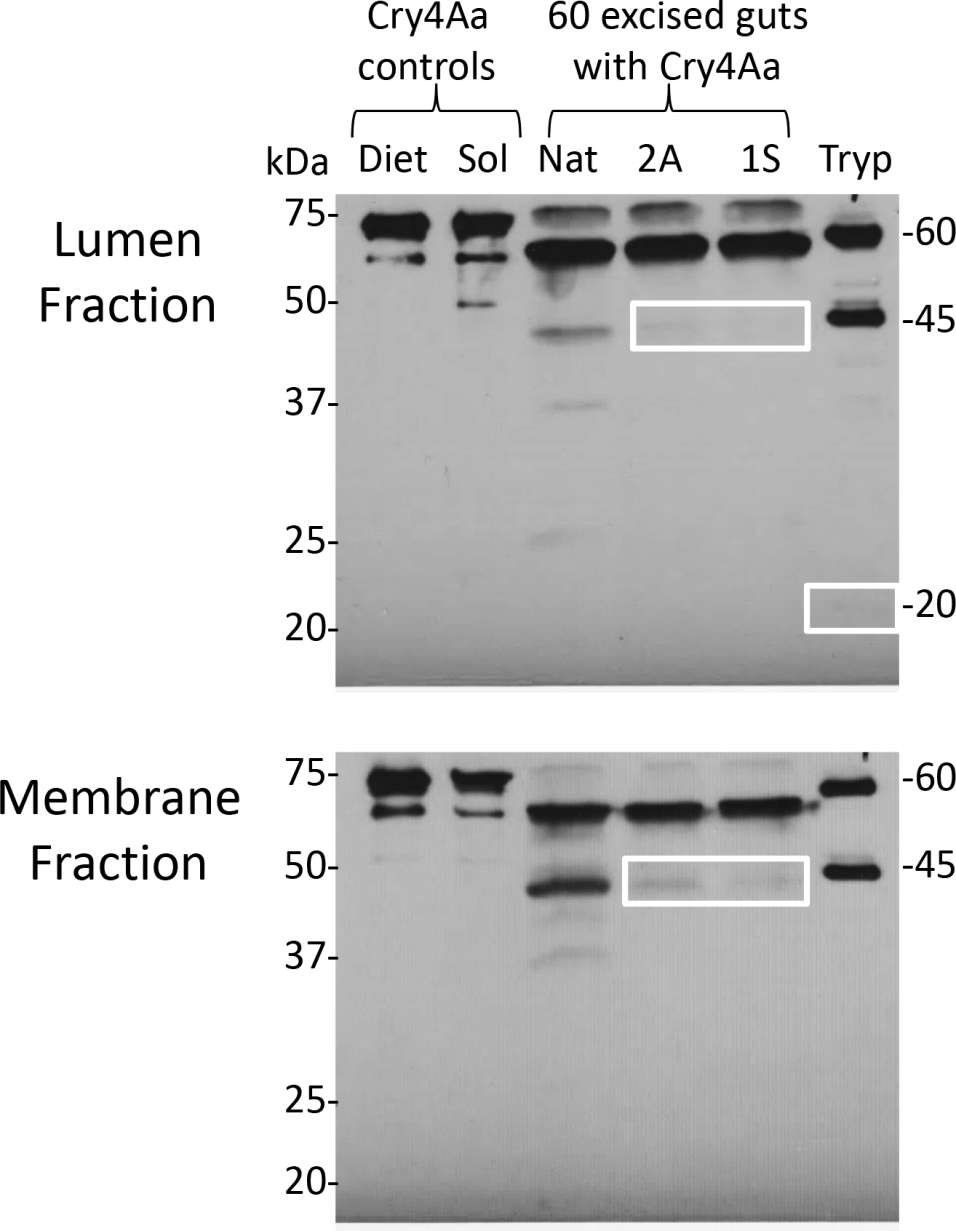
Detection of modified Cry4Aa following exposure to cathepsins in the aphid gut. Aphids were fed overnight, and lumen and membrane fractions from dissected guts were separated by SDS-PAGE and analyzed by western blot for toxin profiles. A second replicate of 57 aphid guts yielded similar results in the lumen fraction. Native Cry4Aa controls: diet—Cry4Aa exposed to aphid diet overnight. Sol, Cry4Aa solubilized in PBS pH 7.4. Tryp, native Cry4Aa exposed to 5% w/w trypsin, (positive control). Nat, native Cry4Aa. Note: none of the controls were exposed to aphid gut proteases. Boxes highlight faint 45 and 20 kDa toxin bands.

Native Cry4Aa in complete aphid diet remained stable with a predominant 65 kDa band, indicating that toxin processing did not occur in aphid diet during the feeding assay (Fig 6). Native Cry4Aa solubilized in 10 mM Tris pH 7.5 resulted in appearance of a faint 50 -kDa band. Trypsin activation of native Cry4Aa resulted in a prominent 45 -kDa band and a faint 20 kDa band (Fig 6).

Gut lumen and membrane fractions prepared from 60 aphids fed native Cry4Aa resulted in digestion of the native Cry4Aa and modified toxins with strong 60 kDa bands apparent in all cases. In contrast to the native Cry4Aa, the 45 kDa protein band resulting from digestion of the modified toxins was more difficult to see on the blot (Fig 6), suggesting that less *in vivo* processing of the modified toxins occurred compared to the native Cry4Aa in either the membrane or the lumen gut protease fractions. The 20 kDa toxin cleavage product was not visible on these western blots.

## Discussion

We introduced cathepsin L and B sites into Cry4Aa-S1 to improve toxin processing within the aphid gut environment. Modified toxins exposed to pea aphid gut lumen proteases, in the presence of protease activators, resulted in increased activation relative to the native Cry4Aa, demonstrating that insertion of cathepsin cleavage sites can be used to facilitate activation *in vitro* (Fig 3). Increased toxin activation in the lumen fraction *in vitro* was observed for all constructs except Cry4Aa 2S. The substitution of amino acids at two sites in Cry4Aa 2S may have interfered with proteolytic cleavage by altering toxin folding, or by interfering with protease accessibility to the cleavage site.

Exposure of native and modified toxin *in vitro* to membrane proteases in the presence of activators did not produce a prominent 45-kDa band. The highest molecular mass toxin band (65 kDa) appears weaker, suggesting degradation of both this and the 45-kDa band. In the absence of activators, protease exposure resulted in non-specific cleavage, as indicated by multiple bands as well as the 45-kDa band (Fig 3). In the aphid gut the majority of proteases are membrane–associated [27]. Although the major proteases present in the aphid gut are cathepsin L and B, other types of protease may be involved with toxin processing. The data shown in Fig 3 suggest that Cry4Aa toxins exposed to membrane proteases are over-digested in the presence of activators (with the exception of the protease-resistant 60 kDa band, see Fig 2A), and hence the lack of the 45-kDa band. In the absence of activators the activity of the membrane proteases is sufficient to cause partial toxin degradation resulting in multiple toxin bands.

Pea aphids experienced increased mortality on day 2 of the bioassay, compared to controls, when exposed to Cry4Aa 2A and trypsin-activated Cry4Aa (Fig 4). The increased toxicity of trypsin activated Cry4Aa against the pea aphid is consistent with the results of Porcar et al. [28] and consistent with the fact that toxin activation by insect proteases is crucial for toxicity [16, 45, 46]. The enhanced processing of Cry4Aa 2A when incubated with lumen proteases *in vitro*, fits with improved toxicity of this construct in bioassays and with toxin activation being crucial for toxicity.

Notably, the other modified Cry4Aa constructs, which also showed increased activation *in vitro*, did not result in enhanced toxicity relative to native Cry4Aa in aphid bioassays. The addition of amino acids, as opposed to substitution of amino acids during modification, would retain all residues which may be crucial for correct folding, toxin association with the insect membrane, and for pore formation. In contrast, substitution constructs lack amino acids that are present in the native toxin that may affect activation. Maintenance of toxicity of all modified constructs to larvae of *C*. *pipiens* indicates that loss of these residues did not interfere with downstream events that are necessary for toxicity.

The feasibility of enhancing toxin activation in less susceptible insect species by introducing sites at the appropriate regions in the toxin has been demonstrated previously [29]. In addition to demonstrating increased toxicity of the modified toxin against western corn rootworm neonates, proteolytic activation also facilitated increased specific binding to western corn rootworm brush border membrane vesicle (BBMV). Walters et al. [29] concluded that the enhanced toxicity was due to the introduction of cleavage sites, which increased activation and subsequent binding to midgut cells. The results from our study on the pea aphid are much less dramatic. By exploiting the major proteases utilized in the aphid gut (cathepsin L and B) and modifying a Cry toxin that, when activated, is toxic against pea aphids we found enhancement of toxin activation *in vitro* (in pea aphid lumen proteases). However, slightly increased toxicity was only observed for one of the modified Cry4Aa constructs, Cry4Aa 2A during in vivo bioassays.

Consistent with the increased aphid toxicity observed for Cry4Aa 2A processing of the toxin was enhanced by lumen proteases relative to processing of native Cry4Aa under *in vitro* conditions with a stronger 45 kDa band apparent (see boxed 45 kDa bands for lumen fraction, Fig 3). The digestion profiles for native Cry4Aa and Cry4Aa 2A on exposure to membrane proteases however were similar. In contrast, Cry4Aa 2A showed only a faint 45 -kDa band *in vivo* in the lumen and membrane fractions of dissected aphid guts, that was less intense than that of the native toxin, suggesting that native Cry4Aa was activated to a greater extent than Cry4Aa 2A. The explanation for this result, which is inconsistent with the *in vitro* data, is unclear.

Modifications made to Cry toxins for enhanced efficacy may interfere with toxin activation either by conformational changes reducing protease accessibility to the cleavage site, or by adjoining residues interfering with protease binding. Protease activity can be influenced not only by the target amino acids but also by adjacent residues. In trypsin proteases the substrate binding site is deep and narrow, with a negatively charged aspartate at the bottom. Cleavage can only occur with amino acids that have long side chains and are positively charged; only arginine and lysine are appropriate for this site [47]. Adjoining residues have the potential to interfere with these stringent requirements and limit protease cleavage. The residues adjoining the inserted cathepsin L/B cleavage sites may also be involved with limiting protease access and result in limiting activation. This scenario seems unlikely for Cry4Aa 2A given the enhanced *in vitro* activation and increased toxicity observed in bioassays.

Among our *in vitro* and *in vivo* experiments we did observe some activation of the native Cry4Aa which is surprising given that cathepsin L and B constitute the major proteases in the aphid gut [23]. There are no other FR cathepsin L cleavage sites in Cry4Aa, while there are two RR sequences (R355 -R356, and R481 -R482) that could be cleaved by cathepsin B. Trypsin-like protease sequences are present in the pea aphid genome and trypsin-like mRNAs have been detected, but it is unknown if these proteases are expressed in the gut [48]. Trypsin-like protease activity was not detected in the pea aphid gut in a previous study [23].

## Conclusions

Previous research has been focused on Cry toxin activation, with susceptible insects cleaving the toxin at specific sites and activation being a precursor to toxicity [16, 46, 49]. Insects that are less susceptible to Cry toxins often lack the proteases required for activation or do not achieve sufficient activation for toxicity [27]. Toxin activation prior to feeding can result in toxicity in these insects [28], suggesting that activation is a rate-limiting step in some less susceptible insects. Our hypothesis that Cry4Aa modified with cathepsin L and B cleavage sites will result in toxin activation was only partially supported by *in vitro* exposure of modified Cry4Aa to aphid gut proteases. However, incorporation of cathepsin cleavage sites was associated with increased toxicity for Cry4Aa 2A. Additional modification of Cry4Aa, such as removal of potential sites involved in degradation or addition of peptides for improved binding to the gut [50], may be required to reach levels of toxicity appropriate for use of modified Cry4Aa in transgenic plants. Expanding the currently used Bt transgenic technology to include toxins active against aphids would facilitate environmentally benign management of these pests.
